# Effect of psychosocial stress and coping strategies on non-surgical periodontal therapy in patients with generalized stage III/IV periodontitis: a longitudinal intervention study

**DOI:** 10.1007/s00784-023-04956-w

**Published:** 2023-03-24

**Authors:** Federica Romano, Ahmad Bebars, Valentina Ortu, Michele Bottone, Marta Giraudi, Giulia Maria Mariani, Giacomo Baima, Mario Aimetti

**Affiliations:** 1 Department of Surgical Sciences, C.I.R. Dental School, Section of Periodontology, University of Turin, via Nizza, 230 Turin, Italy; 2 Private Practice, Turin, Italy; 3 Politecnico di Torino, Turin, Italy

**Keywords:** Coping, Periodontal disease, Periodontal therapy, Psychosocial factors, Stress

## Abstract

**Objectives:**

The aim of this longitudinal intervention study was to assess the impact of psychosocial stress and coping response strategies on the clinical outcomes in periodontitis patients treated with non-surgical periodontal therapy (NSPT).

**Materials and methods:**

After the administration of psychological questionnaires, patients diagnosed with generalized stage III–IV periodontitis were categorized into different groups depending on their stress levels (10-item perceived stress level (PSS-10)) and coping response strategies (coping responses inventory (CRI)). Clinical data were collected 1 week before and 3 months after the completion of NSPT.

**Results:**

Of the 90 patients included at baseline, 27 presented major and 63 minor stress levels, while 40 had avoidance and 50 approach coping behavior. All clinical parameters were similar at the baseline across different categories. At re-evaluation, full-mouth bleeding score (FMBS), mean probing pocket depth (PPD), and number of residual pathological pockets were significantly superior in groups with higher stress levels (*p* <0.001, *p* =0.001, and *p* =0.020, respectively), while higher full-mouth plaque scores (FMPS) and FMBS were found in patients with avoidance coping strategies (*p* =0.009 and *p* <0.001, respectively). When jointly evaluated, an added detrimental effect of coping styles on allostatic load was observed. Multivariate analysis confirmed a significant effect of stress levels and coping strategies on final FMBS, but not of coping on mean PPD.

**Conclusion:**

Psychosocial stress and avoidance coping strategy seem to negatively influence the clinical outcomes of NSPT at short term (NCT04739475; 9/1/2017).

**Practical implications:**

Based on these findings, patients reflecting these psychological profiles should be considered at greater risk for poor NSPT response and may benefit from complementary stress management strategies.

## Introduction

Periodontitis is a dysbiotic biofilm-initiated chronic inflammatory disease of the tooth-supporting apparatus which is mediated by a dysregulated host response leading to a progressive loss of connective attachment and supporting alveolar bone [1]. Current evidence supports multifactorial disease determinants, including smoking, chronic inflammatory comorbidities, genetics, and allostatic stress [2, 3]. The same factors may also influence the response to periodontal treatment; thus, their role should be carefully assessed in the patient risk management and treatment planning [4].

Stress is defined as the psychological response of the organism to a perceived challenge or threat, occurring when an individual perceives that environmental demands exceed his or her adaptive capacity [5]. Psychological stress is an important modifiable risk factor for mental and physical illnesses, through both health-impairing behaviors and pathophysiological pathways [6–8]. Although allostatic load is hard to measure, some instruments have been validated to assess apprehended levels of stress in the widespread population—such as the 10-item perceived stress scale (PSS-10) [9]. Usually, individuals attempt to relieve or cope with perceived stress via two basic patterns: approach and avoidance, depending on their cognitive efforts and abilities [10]. Hence, the potential health impact of stress could be directly related to coping styles that are favorable or detrimental with respect to health outcomes [11].

Periodontitis is a socially patterned condition with a strong behavioral component [12], and a positive epidemiological association with higher stress levels and negative coping strategies was found [13, 14]. Despite this evidence, the influence of these psychosocial factors on periodontal response after non-surgical periodontal therapy (NSPT) has been little investigated [15–17]. In early reports, patients with defensive coping strategies were found to have poorer clinical outcomes after NSPT [17, 18], as well as higher levels of inflammation assessed by plasma cortisol and gingival crevicular fluid (GCF) elastase levels [15, 17]. However, due to the heterogeneity in study design, included population, type of self-administered questionnaires, and absence of adequate control of confounders, there is a need to confirm previous results using reliable measure scales for individuals with periodontitis [19].

Therefore, the aim of this prospective study was to assess the impact of psychosocial stress through PSS-10 tool and coping approach strategies on periodontal inflammation and other clinical periodontal outcomes at 3 months after NSPT, as well as to predict response to treatment using both clinical and psychological variables.

## Materials and methods

This longitudinal intervention study conformed to the ethical guidelines of the 1975 Declaration of Helsinki and was approved by the Ethical Review Board of the A.O.U. Città della Salute e della Scienza di Torino (ID: 0003599, 01/14/21). Prior to enrolment, all patients were informed about the protocol and asked to sign an informed consent. The study protocol has been registered on ClinicalTrials.gov before the start of the trial (ID NCT04739475).

### Study population

Patients seeking treatment in the Section of Periodontology at the University of Turin were consecutively screened as potentially eligible patients for the study from February 2021 to February 2022. Patients fulfilling the following criteria were invited to participate: age > 18 years; presence of ≥ 14 teeth; diagnosis of generalized stage III/IV grade A/B periodontitis with a minimum of 10 sites with probing pocket depth (PPD) ≥ 5 mm [20].

The presence of any of the following excluded a subject from study enrolment: heavy smoker (> 10 cigarettes/die); systemic diseases that could interfere with the clinical response to periodontal treatment (i.e., diabetes mellitus); intake of antibiotics, anti-inflammatory, or psychotropic medications; patients treated by NSPT in the last 6 months prior to recruitment, as well as pregnant women and patients wearing orthodontic appliances.

### Sociodemographic and psychological measurements

At the baseline visit 1 week before initiating NSPT, data on demographic characteristics (gender (man/woman), age (years), education (low: primary and secondary school level; intermediate: high school diploma; high: university degree), smoking behavior (light smoker < 10 cigarettes/day, no smoker)) were collected, and validated questionnaires were administered to all patients. An Italian version of the PSS-10 questionnaire [21] and the Coping Responses Inventory-Adult form (CRI-Adult) [22] were utilized. The PSS-10 is a 10-question self-report questionnaire that measures the amount of stress perceived by an individual. Participants were asked about their feelings and thoughts during the last month using a 5-point Likert scale (never, almost never, sometimes, quite often, and very often). Overall scores are between 0 and 40, with higher values indicating greater stress. Using a median split approach, a point of 20 represents the threshold for minor and major stress levels [23].

CRI identifies the strategies developed by an individual to cope with recent stressful life circumstances. Each coping style has 4 subscales (cognitive and behavioral) measured by 6 items on a 4-point Likert scale from 0 (“not at all”) to 3 (“fairly often”). Patients were categorized into two groups based on the higher CRI score between the two types of coping (approach versus avoidance coping strategy), using the Italian version [24]. A single trained assessor (V.O.) administered all psychological questionnaires, and patients remained unaware of which category of stress they were included until the end of the trial.

### Clinical procedures

The following clinical outcome measures were recorded at six sites for each tooth using a manual periodontal probe (PCP-UNC 15, Hu-Friedy, Chicago, IL, USA*)*: plaque index (PI), gingival recession (Rec), PPD, bleeding on probing (BoP), clinical attachment level (CAL), full-mouth plaque scores (FMPS), and full-mouth bleeding scores (FMBS). After providing personalized oral hygiene instructions regarding brushing technique and the use of interproximal hygiene devices, supragingival scaling was performed, as well as the removal of plaque retentive factors (such as overhanging fillings/crowns or caries). One week later, NSPT was administered by two experienced clinicians following a conventional stage debridement on a quadrant protocol, consisting of 4 appointments at days 0, 7, 14, and 21 [25]. Patients were reviewed at 3 months after the last NSPT appointment when all periodontal clinical parameters were again recorded. A single trained assessor (A.B.), not involved in providing NSPT, performed all clinical measurements. Both the clinicians and the clinical examiner were masked to the results of psychological tests. Before the start of the study, the assessor was calibrated against a reference examiner on 10 non-study subjects, and he was assessed for reproducibility by duplicating periodontal examinations on the same patients within 24 h. The level of agreement with the reference examiner was ≥ 90%, and the intra-examiner reproducibility between the first and the second recording was ≥ 95% using the weighted (± 1 mm) kappa test. Patients not attending to recall or having used antibiotics or anti-inflammatory drugs during follow-up were excluded from the study.

### Data analysis

Statistical analysis was performed using the SPSS Statistics software 25.0 (IBM Corp., Armonk, NY, USA*)*. Categorical data were presented as absolute, and relative frequencies and quantitative data were described as mean and standard deviation (SD). Comparisons of demographic and clinical parameters according to the psychosocial stress level and coping strategy were carried out using chi-square or Fisher’s exact tests for qualitative variables and independent *t*-test or Wilcoxon signed-rank test for quantitative ones as appropriate. Within groups, changes in clinical variables were analysed using the paired *t*-test or the Mann-Whitney *U*-test.

When the subjects were categorised into four groups according to both the stress level and coping strategy, clinical data at baseline and follow-up were analysed using one-way ANOVA or Kruskal-Wallis test, followed by post hoc test. Finally, to assess factors associated with final mean PPD and FMBS%, linear regression models were evaluated with a stepwise backward approach. Selecting potentially statistically (level for entry < 0.25 in the univariate analysis) and clinically significant variables, the significance level was set at 5%.

The number of subjects required was 90 to obtain a difference of 15% in FMBS between minor and major stress levels with a SD of 25%, an alpha error of 5% and a power of 80%, and by adding a 15% in case of possible drop out.

## Results

Table 1 shows the demographic characteristics of the study population. Out of 303 outpatients screened, 90 patients met the inclusion criteria and were enrolled in the study (53 females and 37 males). The mean age was 56.0 ± 10.7 years, with the percentages of light smokers being 6.7%, and 66.7% with low/intermediate education levels. Based on PSS scores, patients were divided in 2 categories: minor (*n* = 63) and major stress level (*n* = 27). Also, the same patients were classified in 2 categories based on CRI scores: approach (*n* = 50) and avoidance (*n* = 40) coping strategy. No statistically significant difference was found for any of the demographic variables at baseline across the different categories.

**Table 1:** Characteristics of the study population at baseline, stratified for stress level and coping behavior

Variables	Minor stress (*n* = 63)	Major stress (*n* = 27)	*p*-value	Approach coping strategy (*n* = 50)	Avoidance coping strategy (*n* = 40)	*p*-value
Age (mean ± SD)	55.4 ± 10.7	57.1 ± 10.8	0.493	54.9 ± 9.9	57.3 ± 11.7	0.299
Sex			0.326			0.848
Male (*n*, %)	28 (44.4)	9 (33.3)		21 (42.0)	16 (40.0)	
Female (*n*, %)	35 (55.6)	18 (66.7)		29 (58.0)	24 (60.0)	
Education level			0.700			0.369
Low (*n*, %)	16 (25.4)	8 (29.6)		13 (26.0)	11 (27.5)	
Intermediate (*n*, %)	27 (42.9)	9 (33.3)		23 (46.0)	13 (32.5)	
High (*n*, %)	20 (31.7)	10 (37.0)		14 (28.0)	16 (40.0)	
Smoking			0.664			0.777
Never (*n*, %)	58 (92.1)	26 (96.3)		47 (94.0)	37 (92.5)	
< 10 cigarettes (*n*, %)	5 (7.9)	1 (3.7)		3 (6.0)	3 (7.5)	
PSS (mean ± SD)	12.7 ± 4.7	26.7 ± 3.5	<0.001	14.9 ± 6.6	18.0 ± 7.2	0.041

Abbreviations: *SD*, standard deviation; *PSS*, perceived stress scale

### Effects of stress level on NSPT outcomes

Table 2 shows the changes in clinical parameters after NSPT according to the stress level category. At baseline, all clinical parameters did not differ between the 2 groups. NSPT was effective in both groups, and statistically significant improvements were found for all clinical parameters. At the re-evaluation, FMPS did not differ between the 2 groups, whereas FMBS resulted significantly more in the minor stress group (*p* <0.001). Accordingly, mean PPD and CAL were higher in the minor stress group (*p* =0.001 and *p* =0.013, respectively), as well as mean number of PPD ≥ 6 mm (*p* =0.020). Conversely, mean number of teeth and PPD 4–5 mm did not show a significant differential change between the groups.

**Table 2:** Changes in clinical parameters (mean ± SD) after non-surgical periodontal therapy according to stress level category

Variable	Baseline			Re-evaluation		
	Minor stress level (*n* = 63)	Major stress level (*n* = 27)	*p*-value	Minor stress level (*n* = 63)	Major stress level (*n* = 27)	*p*-value
N° teeth	26.4 ± 3.2	26.0 ± 2.6	0.338	25.7 ± 3.4**	25.4 ± 2.8*	0.337
FMBS, %	66.1 ± 23.9	69.6 ± 21.6	0.573	20.2 ± 8.2**	39.8 ± 12.8**	< 0.001
FMPS, %	68.7 ± 20.3	69.5 ± 22.0	0.711	20.2 ± 10.2**	24.1 ± 10.9**	0.105
Sites with PPD ≥ 6 mm	26.2 ± 20.8	29.8 ± 24.6	0.815	8.1 ± 6.7**	12.5 ± 8.8**	0.020
Sites with PPD 4–5 mm	48.8 ± 21.6	28.3 ± 18.1	0.771	27.4 ± 16.2**	32.3 ± 15.6**	0.141
Sites with PPD ≤ 3 mm	82.5 ± 29.9	77.0 ± 28.6	0.337	119.2 ± 29.6**	106.8 ± 23.5**	0.012
PPD, mm	3.8 ± 0.9	4.0 ± 0.6	0.193	2.9 ± 0.5**	3.3 ± 0.5**	0.001
CAL, mm	4.4 ± 1.4	4.7 ± 1.3	0.059	3.8 ± 1.0**	4.4 ± 1.2*	0.013

Abbreviations: *CAL*, clinical attachment level; *FMPS*, full-mouth plaque score; *FMBS*, full-mouth bleeding score; *PPD*, probing pocket depth; *SD*, standard deviation

*=*p* <0.05, changes against the baseline; **=*p* <0.001, changes against the baseline

### Effects of coping behavior on NPST outcomes

Table 3 shows the changes in clinical parameters after NSPT according to the coping behavior strategy. At baseline, all clinical parameters did not differ between the 2 groups. Also here, NST was effective in both groups, and statistically significant improvements were found for all clinical parameters. Notably, final FMPS and FMBS were significantly lower in the approach coping group (*p* =0.017 and *p* =0.001, respectively). Conversely, mean number of teeth, mean PPD, CAL, and number of residual pathological sites did not show a significant difference between the groups.

**Table 3:** Changes in clinical parameters (mean ± SD) after non-surgical periodontal therapy according to coping behavior strategy

Variable	Baseline			Re-evaluation		
	Approach strategy (*n* = 50)	Avoidance strategy (*n* = 40)	*p*-value	Approach strategy (*n* = 50)	Avoidance strategy (*n* = 40)	*p*-value
N° teeth	26.5 ± 2.9	26.0 ± 3.1	0.490	25.9 ± 3.2*	25.3 ± 3.2*	0.326
FMBS, %	66.4 ± 24.6	68.0 ± 21.5	0.739	21.7 ± 10.2**	31.6 ± 14.7**	0.001
FMPS, %	69.8 ± 20.5	67.8 ± 21.2	0.679	19.4 ± 10.5**	23.9 ± 10.0**	0.017
Sites with PPD ≥ 6 mm	26.8 ± 21.6	297.9 ± 22.7	0.961	9.7 ± 7.4**	9.1 ± 8.0**	0.539
Sites with PPD 4–5 mm	48.6 ± 21.1	48.7 ± 20.0	0.951	29.3 ± 15.4**	28.4 ± 17.1**	0.620
Sites with PPD ≤ 3 mm	82.5 ± 29.1	78.8 ± 30.2	0.643	116.3 ± 27.7**	114.5 ± 29.5**	0.823
PPD, mm	3.8 ± 0.9	3.9 ± 0.7	0.547	3.0 ± 0.4**	3.1 ± 0.7**	0.907
CAL, mm	4.4 ± 1.4	4.7 ± 1.3	0.256	3.8 ± 1.0**	4.2 ± 1.2*	0.094

Abbreviations: *CAL*, clinical attachment level; *FMPS*, full-mouth plaque score; *FMBS*, full-mouth bleeding score; *PPD*, probing pocket depth; *SD*, standard deviation

*=*p* <0.01, changes against the baseline; **=*p* <0.001, changes against the baseline

### Effects of both stress levels and coping behavior on NSPT

Table 4 shows the changes in clinical parameters after NSPT according to both stress level and coping behavior strategy. According to both PSS and CRI scores, patients were categorized into 4 groups: major stress/avoidance coping (*n* = 17); major stress/approach coping (*n* = 10); minor stress/avoidance coping (*n* = 23); minor stress/approach coping (*n* = 40).

**Table 4:** Changes in clinical parameters (mean ± SD) after non-surgical periodontal therapy according to stress level category and coping response behavior

	Baseline					Re-evaluation				
	Stress + coping + (*n* = 10)	Stress + coping − (*n* = 17)	Stress − coping + (*n* = 40)	Stress − coping − (*n* = 23)	*p*-value	Stress + coping +	Stress + coping −	Stress − coping +	Stress − coping −	*p*-value
N° teeth	25.7 ± 2.3	26.2 ± 2.9	26.7 ± 3.1	25.9 ± 3.3	0.604	25.6 ± 2.2	25.3 ± 3.1	26.0 ± 3.5	25.4 ± 3.3	0.646
FMBS, %	70.9 ± 22.9	68.9 ± 21.4	62.2 ± 25.2	67.4 ± 21.9	0.926	33.2 ± 12.0	43.7 ± 11.9	18.8 ± 7.5^a^	22.6 ± 9.0^a^	<0.001
FMPS, %	72.7 ± 20.1	67.6 ± 23.5	69.1 ± 20.7	67.9 ± 19.9	0.922	16.2 ± 7.4^a^	28.8 ± 10.1	20.2 ± 11.1^a^	20.2 ± 8.5	0.009
Sites with PPD ≥ 6 mm	31.3 ± 22.5	28.9 ± 26.4	25.7 ± 21.5	27.1 ± 20.1	0.867	14.6 ± 6.5	11.2 ± 9.8	8.5 ± 7.1	7.5 ± 6.0^a^	0.047
Sites with PPD 4–5 mm	51.9 ± 15.3	46.2 ± 19.3	47.7 ± 22.4	50.5 ± 20.5	0.686	37.2 ± 13.1	29.4 ± 16.7	27.3 ± 15.5	27.6 ± 17.7	0.216
Sites with PPD ≤ 3 mm	70.9 ± 22.5	80.7 ± 31.7	85.4 ± 30.1	77.5 ± 29.7	0.487	99.6 ± 17.3	111.1 ± 26.1	120.5 ± 28.4^a^	117.1 ± 32.1	0.041
PPD, mm	4.1 ± 0.6	3.9 ± 0.7	3.7 ± 1.0	3.9 ± 0.7	0.505	3.4 ± 0.3	3.3 ± 0.6	2.9 ± 0.4	2.8 ± 0.7^a^	0.007
CAL, mm	4.3 ± 1.5	4.9 ± 1.0	4.4 ± 1.4	4.5 ± 1.4	0.234	4.3 ± 1.0	4.5 ± 1.3	3.7 ± 1.0	4.0 ± 1.1	0.055

Abbreviations: *CAL*, clinical attachment level; *FMPS*, full-mouth plaque score; *FMBS*, full-mouth bleeding score; *PPD*, probing pocket depth; Stress +, major stress level; Stress −, minor stress level; Coping +, approach coping strategy; Coping −, avoidance coping strategy

^a^= significant difference vs stress+/coping−

At baseline, all clinical parameters did not differ between the 4 groups, whereas NSPT was effective for all clinical parameters. As for FMBS, the major stress/avoidance coping group retained a significantly higher number of bleeding sites than minor stress groups, independently from coping attitude (*p* <0.001). As for FMPS, the major stress/avoidance coping group retained a significantly higher number of sites harboring plaque than approach coping groups, independently from stress level (*p* =0.009). Mean PPD and mean number of residual PPD ≥ 6 were significantly higher for the major stress/avoidance group compared to the minor stress/avoidance group (*p* =0.007 and *p* =0.047, respectively). Mean number of teeth and PPD 4–5 mm did not significantly differ at the re-evaluation among the groups.

Table 5 shows a linear regression model considering both FMBS and PPD at the re-evaluation as dependent variables. Major stress level, avoidance coping strategy, number of PPD ≥ 6 mm at baseline, and FMPS at T1 were predictors of mean FMBS at the re-evaluation. On the other hand, major stress level, number of PPD ≥ 6 mm at baseline, and FMPS reduction were positive predictors of mean PPD at the re-evaluation, whereas coping strategy was not related.

**Table 5:** Final linear regression models for final FMBS and PPD as dependent variables

Dependent variable	Estimate	95% CI	*p*-value
Mean FMBS at T1			
Stress level (major/minor)	15.855	11.477; 20.233	<0.001
Coping (approach/avoidance)	−5.160	−9.084; 1.236	0.011
N PPD ≥ 6 mm	0.284	0.028; 0.539	0.030
FMPS at T1	0.289	0.106; 0.472	0.002
Mean PPD at T1			
Stress level (major/minor)	0.407	0.186; 0.628	<0.001
Coping (approach/avoidance)	0.121	−0.084; 0.325	0.244
N PPD ≥ 6 mm	0.011	0.006; 0.015	<0.001
ΔFMPS	−0.005	−0.009; −0.001	0.029

Abbreviations: *FMPS*, full-mouth plaque score; *FMBS*, full-mouth bleeding score; *PPD*, probing pocket depth; T1, 3-month re-evaluation

## Discussion

This study demonstrated the negative impact of psychological factors such as stress and coping behavior on NSPT outcomes in generalized stage III–IV periodontitis, highlighting the importance of psychological evaluation and management as part of the overall patient care. Superior results in terms of FMBS, mean PPD, and number of severe pathological pockets were obtained in the group with minor stress level at 3 months after NSPT, despite similar oral hygiene compliance. Coping strategies alone had a major influence on FMPS and FMBS, but no differences were found regarding other periodontal clinical parameters at the re-evaluation. When stress and coping were jointly assessed, mean PPD and number of residual pockets were significantly higher for the major compared to the minor stress group independently from coping, which seemed to influence more FMPS values through the behavioral component. Multivariate analysis confirmed a significant effect of stress levels and coping strategies on final bleeding scores, but not of coping on final PPD values.

Similar results were reported in an early investigation, suggesting better outcomes in terms of PPD and CAL changes in deep bleeding sites of patients with low level of psychological stress using the PSS-10 scale [15]. However, only 2 pocket sites were examined per patient, and plaque scores were not recorded. Another study showed significant associations between psychological variables and periodontal clinical parameters in a multivariate analysis, showing that increased Depression Anxiety Stress Scale (DASS)-stress score was associated to worsened NSPT outcomes in terms of BoP and mean PPD reduction (*p* <0.05) [17]. Moreover, using the Toulouse Coping Scale (TCS), the authors found an influence of negative coping on BoP and PPD reduction at 6 months. Conversely, no association was found between periodontal parameters and psychosocial stress before and after NST in 30 stage III–IV periodontitis patients using the PSS-10 [26]. In another similar study with a 2-year follow-up, patients with a defensive coping style had statistically significant poorer CAL values (*p* <0.001) compared to patients with other types of coping behavior, although stress levels were not assessed [18]. When comprehensively evaluating stress and coping as in the present study, minor stress levels were found associated to a larger improvement in mean FMBS, PPD, and CAL, with negative coping attitude (avoidance strategy) impacting mostly in patients with major stress levels. These relatively unaligned findings might be ascribed to heterogeneous populations, data handling, and different psychometric tools used. In the absence of a gold standard for measuring stress [12], PSS-10 was adopted here as it represents one of the most reliable psychological questionnaire in the medical field [27–29], and it has received extensive validation when studying the relationships between stress and oral diseases, including periodontitis [19, 30]. On the other hand, CRI is suitable for assessing the coping responses of adults, being utilized by a vast body of research in psychiatric, substance abuse, and medical patient [22, 31].

No differences in periodontal clinical parameters were found at baseline across different groups of stress level and coping behavior. Although seeming to contrast with epidemiologic evidence [32], this can be explained by the high homogeneity at baseline. Indeed, only patients with generalized stage III/IV grade A/B periodontitis were included [20]. As expected, NSPT was found efficient in improving all clinical parameters at 3 months, being comparable with other clinical trials using similar approaches for steps I–II of periodontal therapy [33–37]. However, patients in the major stress level tended to present significantly worse periodontal clinical parameters at the re-evaluation. This negative effect could be attributed to both behavioral and biological causes [32]. Stressed subjects tend to adopt health impairing behaviors, such as smoking, alcohol drinking, and bad eating, while neglecting oral hygiene care [38]. However, patients of the present sample were all non- or light smokers with a low level of alcohol consumption, and the effect of stress was not evident on FMPS values at baseline and follow-up. On the other hand, allostatic load is also known to have detrimental biological effects, encompassing an over-stimulation of both the hypothalamic-pituitary-adrenal and the sympathetic axes, with a chronic release of glucocorticoids and catecholamines [39]. These pathophysiological factors indirectly affect hormonal, inflammatory, and immunological profiles, increasing susceptibility to periodontal infection/inflammation and impairing periodontal wound healing [12, 40, 41]. Interestingly, inferior results regarding both post-treatment FMBS and FMPS values were found in patients with avoidance coping strategies, with no differences in other clinical outcomes. These findings may suggest that coping may act more on the behavioral (poorer oral hygiene) than the biological pathway (bleeding/plaque ratio) [42].

The present study can rely on a large sample size with high homogeneity in disease characteristics and on well-validated psychometric tools. Some limitations should be also acknowledged, such as the relatively short follow-up, the presence of residual confounders in the analysis, and the lack of biological parameters to assess the allostatic load. However, it has been demonstrated that salivary and GCF cortisol levels possess a large circadian variation, and they are not generally effective as biomarkers of chronic stress [12, 43]. Further interventional trials assessing the impact of psychological interventions/therapies need to be conducted to address.

## Conclusions

Major stress levels and avoidance coping behavior negatively influenced NSPT outcomes in generalized stage III–IV periodontitis. While allostatic load had an impact on inflammatory parameters and number of residual pathological pockets, coping style mostly impaired plaque control. Based on these findings, patients with high stress level and negative coping behavior should be considered at greater risk for poor treatment response and for disease progression, with relevant consequences in term of treatment planning and supportive periodontal care.

## Data Availability

The data that support the findings of this study are available from the corresponding author upon reasonable request.
